# Blocking the epithelial-to-mesenchymal transition pathway abrogates resistance to anti-folate chemotherapy in lung cancer

**DOI:** 10.1038/cddis.2015.195

**Published:** 2015-07-16

**Authors:** S-Q Liang, T M Marti, P Dorn, L Froment, S R R Hall, S Berezowska, G Kocher, R A Schmid, R-W Peng

**Affiliations:** 1 Division of General Thoracic Surgery, Inselspital University Hospital Bern, Bern, Switzerland; 2 Department of Clinical Research, Thoracic Surgery Stem Cell Laboratory, University of Bern, Bern, Switzerland; 3 Graduate School for Cellular and Biomedical Sciences, University of Bern, Bern, Switzerland; 4 Institute of Pathology, University of Bern, Bern, Switzerland

## Abstract

Anticancer therapies currently used in the clinic often can neither eradicate the tumor nor prevent disease recurrence due to tumor resistance. In this study, we showed that chemoresistance to pemetrexed, a multi-target anti-folate (MTA) chemotherapeutic agent for non-small cell lung cancer (NSCLC), is associated with a stem cell-like phenotype characterized by an enriched stem cell gene signature, augmented aldehyde dehydrogenase activity and greater clonogenic potential. Mechanistically, chemoresistance to MTA requires activation of epithelial-to-mesenchymal transition (EMT) pathway in that an experimentally induced EMT *per se* promotes chemoresistance in NSCLC and inhibition of EMT signaling by kaempferol renders the otherwise chemoresistant cancer cells susceptible to MTA. Relevant to the clinical setting, human primary NSCLC cells with an elevated EMT signaling feature a significantly enhanced potential to resist MTA, whereas concomitant administration of kaempferol abrogates MTA chemoresistance, regardless of whether it is due to an intrinsic or induced activation of the EMT pathway. Collectively, our findings reveal that a *bona fide* activation of EMT pathway is required and sufficient for chemoresistance to MTA and that kaempferol potently regresses this chemotherapy refractory phenotype, highlighting the potential of EMT pathway inhibition to enhance chemotherapeutic response of lung cancer.

Lung cancer is the most common and deadliest among all malignant tumors, causing over one million deaths world-wide each year.^1^ The two major types of lung cancer are non-small cell lung cancer (NSCLC), accounting for about 80–85% of all lung cancer cases, and small cell lung cancer (SCLC) for about 10%. Chemotherapy represents a frontline treatment for lung cancer in particular for NSCLC that is often diagnosed at an advanced stage.^2^ However, conventional chemotherapeutics often can neither stop tumor growth nor prevent its relapse due to tumor resistance to chemotherapy. The molecular mechanisms underlying this phenomenon remain poorly defined,^3^ highlighting an urgent need to understand the cellular and molecular determinants that drive and sustain chemoresistance, which might hold the promise for identification of tumor- and drug-specific alterations that are amenable to molecularly targeted intervention, and for generation of biomarker profiles that will enable personalized therapy.

Experimental and clinical evidence has revealed that cancer cells are heterogeneous regarding tumor-propagating capacity and response to therapeutic drugs. A prevailing hypothesis states that a phenotypically and functionally distinct subpopulation within the tumor, referred to as cancer stem cells (CSCs), dictates tumor propagation and progression and might additionally account for the tumor resistance to therapeutics.^4, 5^ The CSC concept explains plausibly the inefficiency of chemotherapeutic drugs used today and implies that CSCs must be taken into account for effective anticancer strategies aimed at permanent clinical remission of tumors. Supporting this model, tissue-specific CSCs, characterized by a gene signature reminiscent of embryonic stem cells, for example, elevated levels of Sox2, Oct4 and Nanog, and the potential to self-renew and differentiate into multilineage cancer cell types, have been identified in leukemia and solid tumors.^6, 7, 8, 9^ CSCs in some cancers have also been connected with tumor resistance to chemo-, radio- and molecularly targeted therapies.^10, 11, 12^ In NSCLC, several studies have reported the identification of CSCs, based primarily on the expression of cell-surface markers,^13, 14, 15, 16, 17^ and a link between CSCs and NSCLC resistance has also been proposed.^14, 15, 17, 18, 19, 20^

Epithelial-to-mesenchymal transition (EMT) is a trans-differentiation program essential for numerous developmental processes during embryogenesis, enabling epithelial cells to lose cell polarity and cell–cell adhesion and to concomitantly attain mesenchymal characteristics, such as enhanced migration and invasion.^21^ EMT can be triggered by diverse extracellular stimuli, for example, transforming growth factor-*β* (TGF-*β*). As response, recipient cells mobilize a signaling cascade, leading to increased expression of a panel of EMT-inducing transcription factors (EMT-TFs), most prominently *SNAI1/SNAIL1*, *SNAI2/SNAIL2* (also known as *SLUG*), *ZEB1* and *ZEB2*. Upregulation of EMT-TFs in turn modulates the expression of their downstream target genes, which ultimately executes the activation of an EMT.^22, 23^ Remarkably, recent studies have revealed a key role for EMT in potentiation of stem cell features, acquisition of an un-differentiated state and promotion of tumor progression and metastasis.^24, 25^ Furthermore, an important role for EMT in tumor resistance to anticancer therapies has increasingly been appreciated.^26, 27, 28^

In this study, we have investigated chemoresistance to pemetrexed, a multitargeted anti-folate (MTA) chemotherapeutic agent used in the clinic for treatment of NSCLC and malignant pleural mesothelioma (MPM). MTA is analogous to folic acid, a physiological precursor required by both normal and tumor cells for *de novo* purine and pyrimidine biosynthesis.^29^ We show that in NSCLC chemoresistance to MTA is linked to a stem cell-like phenotype and functionally driven by an escalated EMT signaling. We further demonstrate that kaempferol potently regresses this chemotherapy refractory phenotype. Kaempferol is a natural flavonoid existing in many dietary plant sources and previous studies have shown that kaempferol possesses multifaceted biological and pharmacological properties including anticancer properties.^30, 31, 32^ The molecular mechanisms underlying the activities of kaempferol remain largely undefined. To our best knowledge, this is the first report showing that chemoresistance to MTA is attributed to an activated EMT pathway and that kaempferol abrogates this phenotype. Thus, blocking EMT signaling might be a rational strategy to enhance cancer response to chemotherapeutics.

## Results

### Chemoresistance to MTA is associated with a stem cell-like phenotype

To assess how NSCLC responds to conventional chemotherapeutic agents, a panel of NSCLC cell lines (A549, H358 and H460) were treated with pemetrexed (MTA). Cell growth and drug efficacy were subsequently determined by XTT assay according to the NCI60 platform protocol.^33^ MTA treatment imposed a proliferation arrest in A549, H358 and H460 cells (Supplementary Figure S1). Notably, a variable but substantial subpopulation in all three NSCLC lines still proliferated in the presence of MTA (Supplementary Figure S1) and this proliferative potential persisted even at the highest tested drug doses and after prolonged exposure (up to 7 days), suggesting that this fraction of tumor cells could escape the treatment and were MTA resistant.

**Figure 1:**
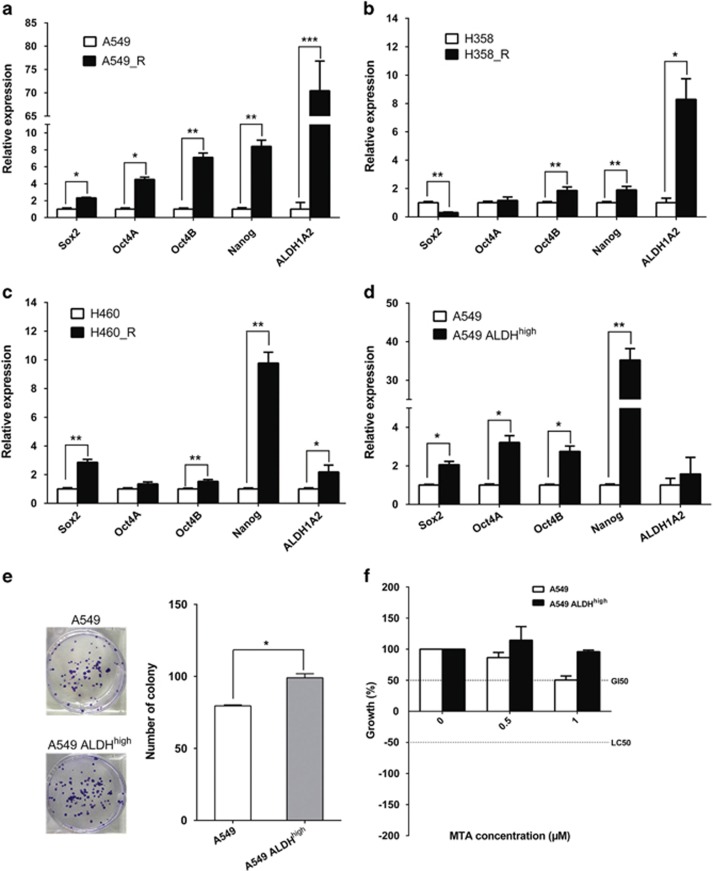
Chemoresistance to MTA is associated with an enriched stem cell gene signature and augmented ALDH activity. (**a–c**) mRNA levels of stem cell genes in chemoresistant A549_R, H358_R, H460_R and their parental A549, H358 and H460 cells were analyzed by qPCR. Data are shown as mean±S.D. of three independent experiments (*n*=3). (**d–f**) Augmented ALDH activity hallmarks a stem cell-like population with enhanced chemoresistance to MTA. The FACS-sorted ALDH^high^ subset and unsorted A549 cells were analyzed by qPCR (**d**), by clonogenic assay (**e**) and by XTT assay after treatment with MTA for 24 h (**f**). Results are presented as mean±S.D. of three independent experiments (*n*=3). For clonogenic assay (**e**), the ALDH^high^ and A549 cells were seeded in six-well plates (250 cells/well) and cultured for 10 days. *P<0.05; **P<0.01; ***P<0.001

To characterize MTA-resistant NSCLC cells, we generated chemoresistant cell lines (A549_R, H358_R and H460_R) by continuously exposing A549, H358 and H460 cells to increasing doses of MTA, starting from the IC_50_ and doubling the MTA concentration every week until a dose of 5–10 × IC_50_ was reached. Gene-expression analysis with quantitative real-time PCR (qPCR) showed that these MTA-resistant cells, irrespective of the NSCLC subtype from which their parental cells were derived, unanimously featured a stem cell-like gene signature characterized by elevated mRNA levels of embryonic stem cell factors Sox2, Oct4 and Nanog (Figure 1a–c), although to a varying extent. Specifically, all three chemoresistant lines (A549_R, H358_R and H460_R) expressed significantly higher levels of Oct4B and Nanog than their respective parental cells (A549, H358 and H460). Significant upregulation of Oct4A was observed in A549_R compared with that in A549 cells. Interestingly, Sox2 was significantly increased in A549_R and H460_R cells but reduced in H358_R cells compared with that in their corresponding parental cells (Figure 1a–c).

To test whether chemoresistance is linked to the acquisition of a stem cell phenotype, we next assessed whether MTA treatment induces clonogenicity in NSCLC cells. Acute treatment (up to 12 h) with MTA indeed promoted clonogenic potential in A549 and H460 cells in a time-dependent manner, as the cells exposed to MTA (5 *μ*M) for prolonged periods gave rise to more colonies than those treated for shorter time (Supplementary Figures S2A and B). MTA (1 *μ*M) also induced clonogenicity in H358 cells, but 6-h treatment induced more colonies than 12-h treatment (Supplementary Figure S2C).

### Augmented ALDH activity marks a stem cell-like subset in NSCLC displaying enhanced MTA resistance

Aldehyde dehydrogenase (ALDH) is a marker of normal and malignant stem cells in a variety of tissues including the lung.^34, 35, 36^ Notably, *ALDH1A2* was among the most prominently upregulated genes in MTA-resistant cells (Figure 1a–c). Consistent with this finding, acute treatment with MTA (10 μM for 24 h) significantly increased the ratio of ALDH-positive cells (Aldefluor assay) in A549 (from 0.86 to 14.7%), H358 (from 1.7 to 15.2%) and H460 cells (from 7.23 to 13%), as indicated by flow cytometry (Supplementary Figure S3A). Further analysis revealed that ALDH-positive cells (ALDH^high^), fractionated by fluorescence-activated cell sorting (FACS; Supplementary Figure S3B), featured a more pronounced stem cell phenotype, manifested by elevated mRNA levels of stem cell factors (Figure 1d; Supplementary Figures S3C and E) and enhanced clonogenic potential (Figure 1e; Supplementary Figures S3D and F) in the ALDH^high^ cells compared with their corresponding parental A549, H358 and H460 cells. Importantly, the ALDH^high^ cells displayed a greater chemoresistance to MTA than the parental cells (Figure 1f). Further supporting that augmented ALDH activity is a feature of stem cells and chemoresistance in NSCLC, *in silico* analysis^37^ of 1570 NSCLC patients showed that high expression of ALDH1A2, but not of ALDH1A1 and ALDH1A3, was associated with poorer prognosis (Supplementary Figure S3G).

### Chemoresistance to MTA requires an activated EMT pathway

**Figure 2:**
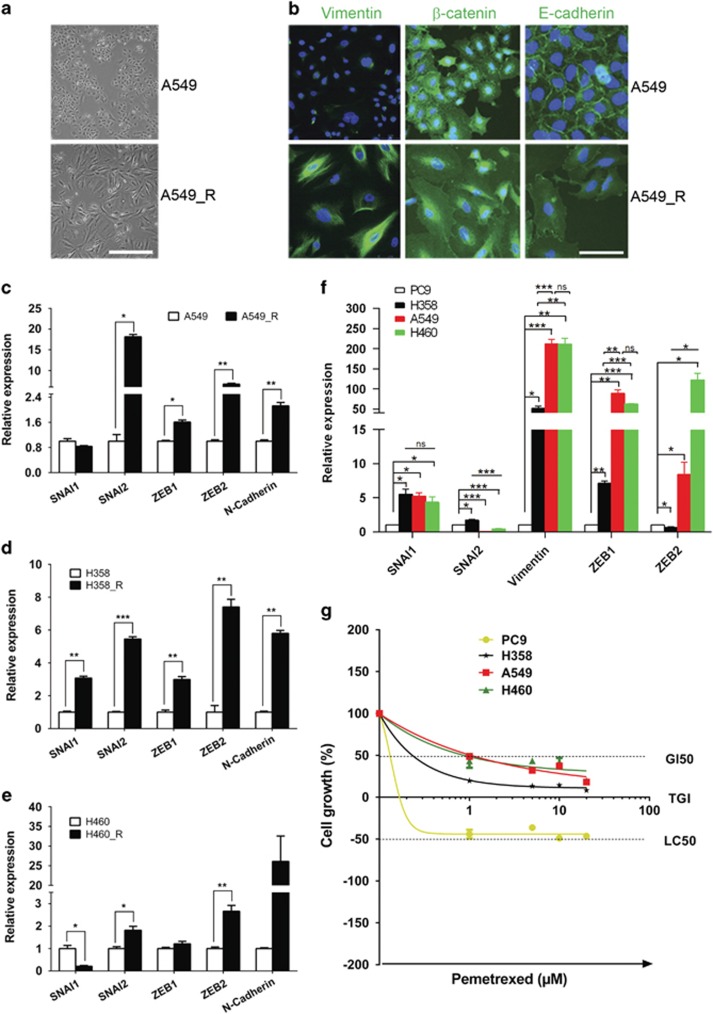
Chemoresistant NSCLC cells exhibit an activated EMT pathway. (**a**) Morphological images of chemoresistant A549_R and parental A549 cells. Scale bar, 100 *μ*m. (**b**) A549_R and A549 cells were immunostained for Vimentin, *β*-catenin and E-cadherin (green). Nuclei were counterstained with DAPI (blue). Scale bar, 25 *μ*m (**c–e**) mRNA levels of EMT-TFs and *CDH2/N-cadherin* in A549_R, H358_R and H460_R cells and the corresponding parental cells (A549, H358 and H460) were analyzed by qPCR. Data are shown as mean±S.D. of three independent experiments (*n*=3). (**f**) Different NSCLC cells are at distinct stages along the EMT. mRNA levels of EMT-TFs and *Vimentin* in PC9, H358, A549 and H460 cells were analyzed by qPCR and results are shown in mean±S.D. of three independent experiments (*n*=3). (**g**) NSCLC cells at distinct EMT stages exhibit differential sensitivity to MTA. PC9, H358, A549 and H460 cells treated with the indicated MTA doses for 5 days were analyzed by XTT assay. The results are shown as mean±S.D. of triplicate experiments. *P<0.05; **P<0.01; ***P<0.001

MTA-resistant NSCLC cells adopted a mesenchymal appearance instead of the epithelial morphology of their parental cells (Figure 2a; Supplementary Figures S4A and B), which prompted us to address the possibility that these cells had undergone an EMT. Immuofluorescence analyses confirmed that E-cadherin and *β*-catenin, two epithelial markers localized at cell–cell adhesion and expressed at high levels in parental A549 cells, were remarkably reduced in chemoresistant A549_R cells (Figure 2b). In contrast, Vimentin, a marker of mesenchymal cells that was marginally detectable in parental A549 cells, was highly abundant in A549_R cells (Figure 2b). Western blot analyses confirmed the change of E-cadherin and Vimentin in A549_R and A549 cells and the same pattern of E-cadherin and Vimentin expression were found in H358_R and H358 (Supplementary Figure S4C). Similarly, H460_R expressed higher level of Vimentin than H460 cells (Supplementary Figure S4C). We did not detect E-cadherin in H460 cells (Supplementary Figure S4C), which is, however, consistent with an earlier study.^26^ In concordance with the immunofluorescence and Western blot results, qPCR analyses revealed that the majority of EMT-TFs (*SNAI1*, *SNAI2*, *ZEB1* and *ZEB2*) and also *CDH2* (*N-cadherin*), another mesenchymal marker, were significantly upregulated in A549_R, H358_R and H460_R cells compared with the parental cells (Figures 2c and e). Moreover, a scratch wound-healing assay indicated that A549_R and H358_R displayed higher mobility than their parental cells (Supplementary Figures S4D and E), consistent with a scenario that EMT is activated in MTA-resistant cells.

The EMT pathway is a dynamic process that consists of serial intermediate stages, by which epithelial cells transition to a mesenchymal state.^23, 38^ We asked whether an intrinsic EMT status is related to chemoresistant potential in NSCLC cells. Analyses with qPCR revealed that EMT-TFs (*SNAI1*, *SNAI2*, *ZEB1* and *ZEB2)* and *Vimentin* were differentially expressed in the NSCLC cell lines A549, H358, H460 and PC9 (Figure 2f; Supplementary Table S1). Significantly, *SNAI1* was highly abundant in H358, A549 and H460 cells compared with that in PC9 cells. Similarly, *ZEB1* and *Vimentin* were expressed at the highest level in A549 and H460 cells, to a lesser extent in H358 cells but only at basal level in PC9 cells (Figure 2f; Supplementary Table S1). Importantly, NSCLC cells that contained more mesenchymal-like subpopulations, for example, A549 and H460 cells, based on the mRNA level of EMT-TFs and *Vimentin*, featured a greater chemoresistance to MTA (Figure 2g). Specifically, A549 and H460 cells that had the highest *ZEB1* and *Vimentin* levels displayed the greatest resistance to MTA. In contrast, PC9 cells that expressed the lowest level of EMT-TFs and *Vimentin* were highly sensitive to MTA treatment (Figure 2g).

**Figure 3:**
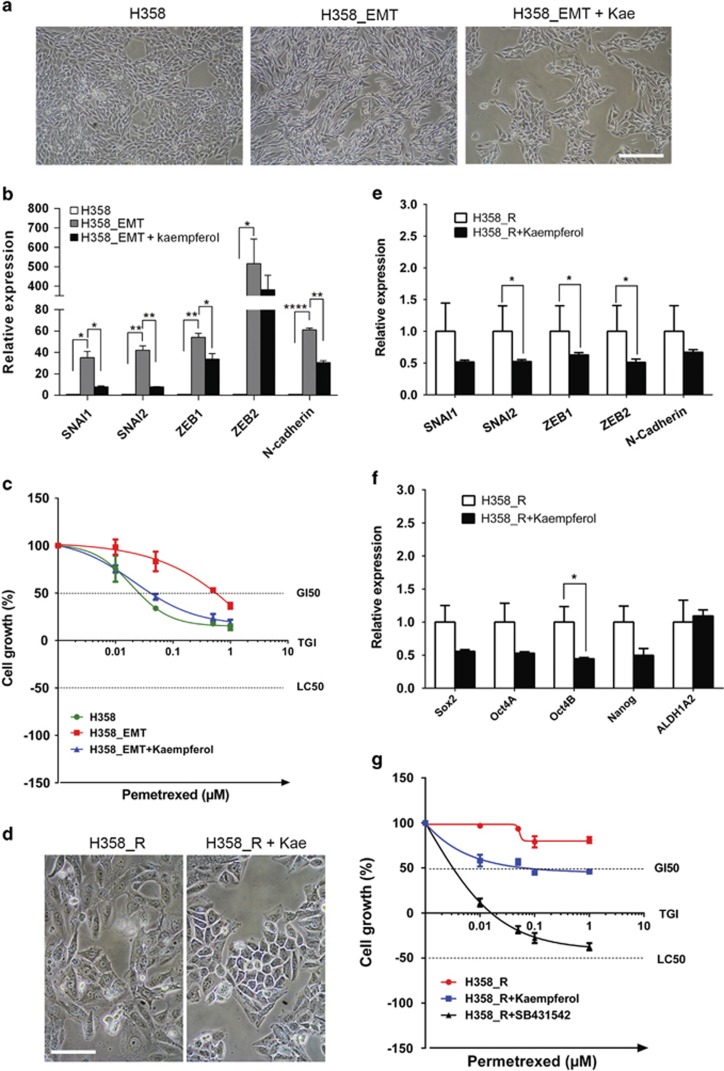
An activated EMT pathway is required for chemoresistance to MTA. (**a**) TGF-*β* induces a morphological switch from epithelial-to-mesenchymal phenotype and kaemperol reverses the process. H358, untreated H358 cells; H358_EMT, H358 cells treated with TGF-*β* (5 ng/ml) for 14 days; H358_EMT+Kae, H358_EMT cells treated with kaempferol (20 *μ*M) for 5 days. Scale bar, 100 *μ*m. (**b**) TGF-*β* treatment activates the EMT pathway whereas kaempferol inhibits the process. The mRNA level of EMT-TFs and *N-cadherin* in H358, H358_EMT and kaempferol-treated H358_EMT cells (H358_EMT+kaempferol; as above) were analyzed by qPCR. Data are shown as mean±S.D. of three independent experiments (*n*=3). (**c**) Chemoresistance to MTA is promoted by an activated EMT pathway and regressed by kaempferol. H358, H358_EMT and kaempferol-treated H358_EMT cells (H358_EMT+kaempferol) were treated with MTA for 5 days and analyzed by XTT assay. Data are shown as mean±S.D. of three independent experiments (*n*=3). (**d**) Micrographic images of chemoresistant H358_R cells treated with vehicle (H358_R) or with 20 *μ*M kaempferol for 3 days (H358_R+kaempferol). Scale bar, 50 *μ*m (**e** and **f**) Kaempferol treatment inhibits the expression of EMT-TFs and stem cell genes. The mRNA level of EMT-TFs and *N-cadherin* (**e**) and of stem cell genes (**f**) in vehicle- and kaempferol-treated H358_R cells (as above) was analyzed by qPCR. Data are shown as mean±S.D. of three independent experiments (*n*=3). (**g**) Kaempferol and SB431542 abrogate chemoresistance to MTA. H358_R cells treated with vehicle, kaempferol (20 *μ*M) and SB431542 (40 *μ*M) for 3 days were then exposed to MTA for another 5 days. Drug response was determined by XTT assay and data are shown as mean±S.D. of three independent experiments (*n*=3). *P<0.05; **P<0.01; ****P<0.001

To address whether EMT is functionally important for chemoresistance, we treated NSCLC cells with TGF-*β* that is known to induce EMT.^24^ TGF-*β* treatment triggered a morphological switch from epithelial-to-mesenchymal phenotype in H358 cells (Figure 3a; Supplementary Figure S5) and qPCR analyses revealed an EMT-TF expression profile consistent with an activated EMT pathway in TGF-*β*-treated H358 and A549 cells (Figure 3b; Supplementary Figure S6A). In concordance with the previous report,^24^ TGF-*β* also potentiated the stem cell phenotype, for example, increased clonogenicity and sphere-forming potential (Supplementary Figures S6B, C and D). Importantly, TGF-*β*-treated H358 and A549 cells that featured an activated EMT pathway were significantly more resistant to MTA than untreated cells (Figure 3c; Supplementary Figure S6E). In detail, the GI50 (50% growth inhibition) concentration of MTA increased from about 0.025 *μ*M (untreated H358 cells) to around 0.6 *μ*M (H358 cells treated with TGF-*β*).

### Kaempferol regresses MTA resistance by inhibiting the EMT pathway

To address whether an activated EMT pathway drives chemoresistance in NSCLC, we performed loss-of-function analyses by treating chemoresistant H358 cells with kaempferol, a natural flavonoid existing in dietary plants and recently identified as an EMT inhibitor.^39^ Confirming the previous report,^39^ kaempferol treatment induced a morphological transition from mesenchymal-to-epithelial phenotype in H358_EMT cells (Figure 3a; Supplementary Figure S5), generated by treating H358 cells with TGF-*β*, and in MTA-resistant H358_R cells (Figures 1b, 2d and 3d; Supplementary Figure S7A). Consistent with the observed morphological switch, kaempferol significantly reduced the expression of EMT-TFs in both H358_EMT and H358_R cells (Figures 3b and e) and concomitantly downregulated the stem cell genes in H358_R cells (Figure 3f). Importantly, kaempferol potently sensitized the chemoresistant H358_EMT and H358_R cells to MTA treatment (Figures 3c and g). Similar effects of kaempferol on chemoresistant A549_R and H460_R were also observed (Supplementary Figure S7B).

TGF-*β* signaling is initiated when TGF-*β* binds its receptors (T*β*RI/ T*β*RII), which can be blocked by SB431542, an inhibitor of T*β*RI.^40^ To dissect whether EMT is directly involved in MTA resistance, H358_R, A549_R and H460_R were analyzed for their response to MTA with or without SB431542. Strikingly, concomitant treatment with SB431542 strongly regressed the resistance of all three cell lines to MTA, albeit to a varying extent (Figure 3g; Supplementary Figure S7B). Thus, chemoresistance to MTA requires a *bona fide* activation of EMT and pharmacological suppression of EMT regresses MTA resistance.

### An activated EMT pathway is required for chemoresistance to MTA in primary NSCLC and pharmacological suppression of EMT enhances tumor response to MTA

**Figure 4:**
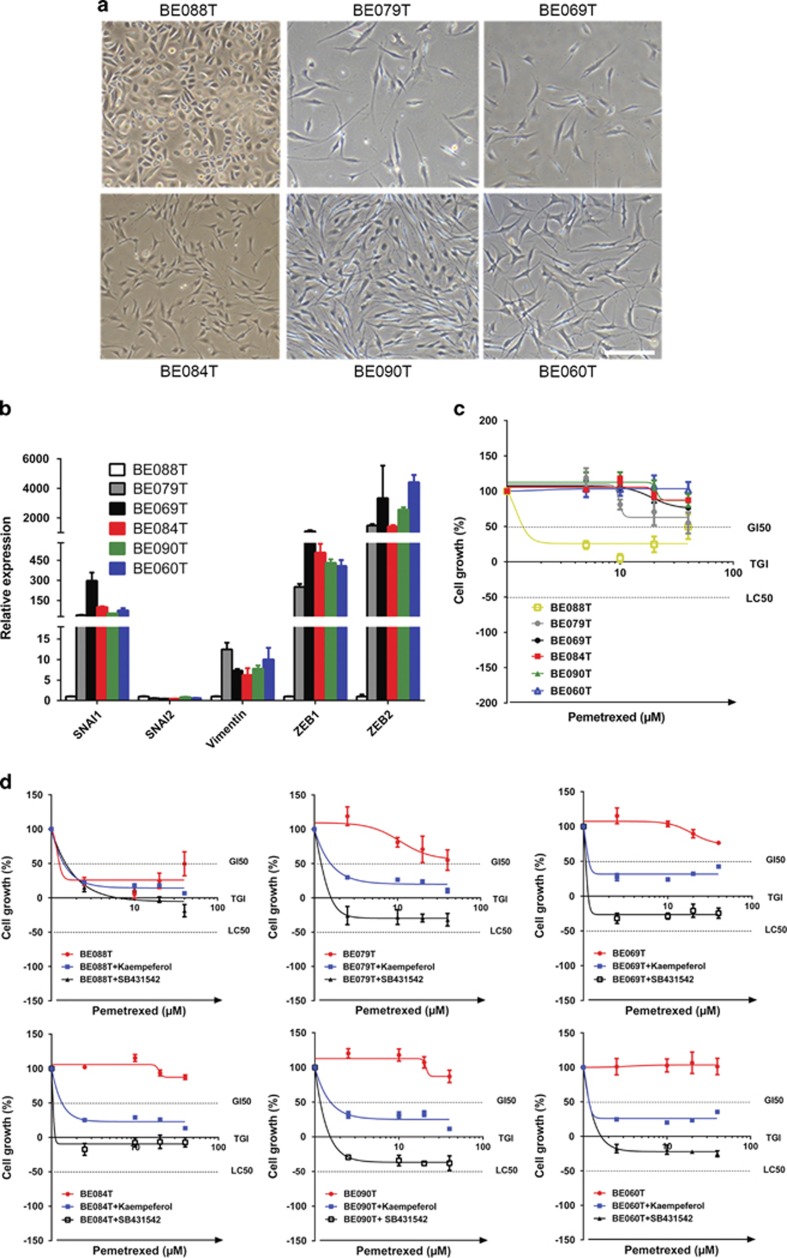
An activated EMT pathway promotes chemoresistance in primary NSCLC cells and kaempferol enhances chemotherapeutic response to MTA. (**a**) Micrographic images of the primary NSCLC cells used in this study. Scale bar, 100 *μ*m. (**b**) The EMT pathway is differentially activated in primary NSCLC cells. The mRNA level of EMT-TFs and *Vimentin* was analyzed by qPCR. (**c**) Primary NSCLC cells exhibit differential sensitivity to MTA. The cells were treated for 5 days with various MTA doses and drug response was determined by XTT assay. (**d**) Kaempferol and SB431542 enhance chemotherapeutic response of primary NSCLC cells to MTA. The primary NSCLC cells treated for 5 days with vehicle, kaempferol (40 *μ*M) or SB431542 (40 *μ*M) were subsequently exposed to MTA for an additional 5 days. Drug response was determined by XTT assay. All the results are shown as mean±S.D. of triplicate experiments

To test the clinical relevance of our findings, we analyzed a panel of primary NSCLC cells generated directly from tumors of NSCLC patients (Supplementary Table S2; Supplementary Figure S8). These primary NSCLC cells were different in morphology, ranging from a typical epithelial appearance, for example, BE088T cells, to a more mesenchymal-like phenotype such as BE090T and BE060T cells (Figure 4a). Paralleling our findings in NSCLC cell lines (Figure 2f), EMT-TFs and *Vimentin* were differentially expressed in these cells, for example, *SNAI1*, *ZEB1/2* and *Vimentin* were expressed at low levels in BE088T but highly abundant in others (BE060T, BE069T, BE079T, BE084T and BE090T), stratifying the cells into two categories: one with basal EMT signaling and the other with an more activated EMT, indicative of more mesenchymal-like subpopulations within the cells (Figure 4b; Supplementary Table S3). Similar to our findings with NSCLC cell lines (Figure 2f), the primary NSCLC cells with more mesenchymal-like subpopulations, for example, BE060T and BE090T cells, displayed much greater chemoresistance to MTA, whereas the cells in which the EMT pathway was not activated, such as BE088T cells, were highly sensitive to MTA (Figure 4c). Importantly and in concordance with the findings in NSCLC cell lines (Figure 3), treatment with kaempferol abrogated MTA resistance in primary NSCLC cells (Figure 4d). Strengthening the finding with NSCLC cells (Figure 3g; Supplementary Figure S7B), SB431542 potently abrogated the resistance of the primary NSCLC cells to MTA (Figure 4d). As an anticipated exception, BE088T were not MTA resistant, thus irrespective of the addition of kaempferol or SB431542 (Figure 4d).

**Figure 5:**
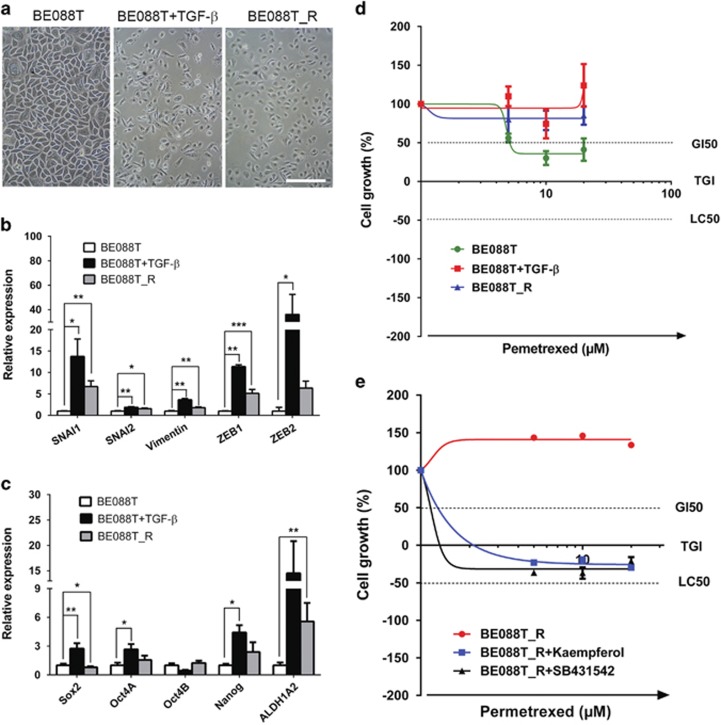
An activated EMT pathway is required for chemoresistance to MTA in primary NSCLC and kaemperol abrogates MTA resistance. (**a**) Micrographic images of primary BE088T cells (BE088T), BE088T cells treated for 14 days with 5 ng/ml TGF-*ββ* (BE088T+TGF-*β*) or with 5 *μ*M MTA (BE088T_R). Scale bar, 100 *μ*m. (**b** and **c**) Treatment with TGF-*β* and MTA activates the EMT pathway and induces stem cell characteristics. BE088T, TGF-*β*- and MTA-treated BE088T cells, as described in (**a**), were analyzed by qPCR for EMT-TFs and *Vimentin* (**b**) and stem cell genes (**c**). Data are shown as mean±S.D. of three independent experiments (*n*=3). (**d**) An activated EMT pathway promotes chemoresistance to MTA. BE088T, TGF-*β*- and MTA-treated BE088T cells (as above) were exposed to MTA for 5 days and analyzed by XTT assay. Data are shown as mean±S.D. of three independent experiments (*n*=3). (**e**) Kaempferol and SB431542 abrogate MTA resistance in primary NSCLC cells. BE088T_R cells were treated with vehicle, kaempferol (40 *μ*M) or SB431542 (40 *μ*M) for 5 days. The resulting cells (BE088T_R, BE088T_R+kaempferol and BE088T_R+SB431542, respectively) were subsequently exposed to MTA for an additional 4 days. The results are shown as mean±S.D. of triplicate experiments. *P<0.05; **P<0.01; ***P<0.001

Finally, we investigated whether an experimentally induced activation of the EMT pathway promotes MTA resistance in primary NSCLC cells. TGF-*β*-treated BE088T cells (BE088T+TGF-*β*) and chemoresistant BE088T_R cells, generated by chronically treating BE088T cells with MTA, featured an activated EMT pathway (Figures 5a and b; Supplementary Figure S9), a more pronounced stem cell-like phenotype (Figure 5c) and, as expected, an enhanced chemoresistance to MTA (Figure 5d) compared with the parental BE088T cells. Importantly, both kaempferol and SB431542 sensitized the otherwise chemoresistant BE088T_R cells to MTA, as concomitant treatment with MTA and kaempferol or SB431542 abolished chemoresistance of BE088T_R cells (Figure 5e).

## Discussion

Despite recent advances in analyses of skin and intestine cancers showing that *bona fide* primary tumors are indeed *de novo* seeded and propagated by CSCs in their natural environment,^8, 9^ there is still a paucity of data that have unequivocally addressed the role and underlying mechanisms for CSCs in therapeutic resistance. Chemotherapy represents a standard option of NSCLC treatment and chemoresistance significantly influences the clinical outcome. In this study, we show that NSCLC resistance to chemotherapeutic MTA is associated with a stem cell-like phenotype, manifested by an increased level of stem cell factors (Sox2, Oct4 and Nanog) and a greater clonogenicity. The involvement of stem cell-like cancer cells in chemoresistance is reinforced by the finding that *ALDH1A2* is one of the most upregulated genes in chemoresistant cells and that the NSCLC subset with an augmented ALDH activity (ALDH^high^) features a greater chemoresistance. We further reveal that an activated EMT pathway is functionally required and sufficient for NSCLC chemoresistance to MTA and that kaempferol potently regresses this chemotherapy refractory phenotype by reversing EMT. Thus, blocking the EMT pathway by kaempferol might be a rational strategy to enhance chemotherapeutic response of lung cancer.

MTA inhibits *de novo* purine and pyrimidine biosynthesis and treatment with MTA induces the expression of thymidylate synthase, a key enzyme in the biosynthetic pathway, which might affect tumor response to MTA.^41^ Recent studies with MPM have implicated mesothelioma cells with stem cell properties, such as high sphere-forming and tumor-initiating capacity, in chemoresistance to MTA.^42, 43^ Consistent with the findings in MPM, we now demonstrate that chemoresistance to MTA in NSCLC is also linked to a stem cell-like phenotype, evidenced by an enriched stem cell gene signature and enhanced clonogenic potential in MTA-resistant cells compared with those in bulk tumor cells (Figure 1 and Supplementary Figure S2)

It has been shown in a variety of malignant tumors including lung cancer that ALDH is a CSC marker.^34, 35^ We demonstrate in this study that the NSCLC subset with an augmented ALDH activity (ALDH^high^) indeed possesses an elevated level of stem cell genes, an elevated clonogenic capacity and, importantly, a greater chemoresistance to MTA (Figure 1). Although we can not exclude the possibility that increased ALDH activity following MTA treatment (Supplementary Figure S3A) might also be contributed by other ALDH isoforms, *ALDH1A2* is, however, one of the most upregulated genes associated with chemoresistance (Figure 1). In contrast, other isoforms,for example, *ALDH1A1*, *ALDH1A3*, *ALDH2* and *ALDH3*, are not significantly altered in chemoresistant cells (data not shown). The importance of *ALDH1A2* in chemoresistance is further supported by our finding that treatment with TGF-*β*, which induces an activated EMT pathway and promotes chemoresistance, also increases *ALDH1A2* expression (Figure 5c). Finally, an *in silico* analysis of a cohort of 1570 NSCLC patients reveals that high expression of *ALDH1A2*, but not of *ALDH1A1* or *ALDH1A3*, is associated with poorer prognosis (Supplementary Figure S3G). Interestingly, a recent report has identified *ALDH1A3* as a key marker of a self-renewing population in NSCLC.^36^ Future studies will be necessary to delineate whether different ALDH isoforms have distinct regulatory roles in cancer.

We demonstrate that chemoresistance to MTA requires an activated EMT pathway. Compelling evidence supporting this conclusion comes from the findings that chemoresistant NSCLC cells feature an activated EMT signaling (Figure 2) and that an experimentally induced EMT *per se* (by treating with TGF-*β*) effectively promotes the acquisition of chemoresistance in both established NSCLC cell lines and primary NSCLC (Figures 3 and 5). Moreover, NSCLC cells with different mRNA levels of EMT-TFs and *Vimentin*, differ in drug response to MTA, with the cells that express higher EMT-TFs and *Vimentin*, display higher chemoresistance (Figures 2f and g 4b and c and Supplementary Table S1). Finally, SB431542, an inhibitor of T*β*RI kinase,^40^ suppresses the resistance to MTA of NSCLC cells (Figures 3g, 4d and 5e; Supplementary Figure S7B), confirming that T*β*RI-mediated EMT is indeed required for NSCLC resistance to MTA. Similar findings have been reported in colorectal and ovarian cancers,^27, 28^ in which chemo- and radiotherapy induces an EMT, and also in NSCLC, where tumor sensitivity to tyrosine kinase inhibitors is dependent on the EMT pathway.^26^

The finding that an activated EMT pathway drives chemoresistance to MTA provides the conceptual possibility for rational design of therapeutic interventions, aimed to overcome this refractory phenomenon of tumors. In light of this context, we showed that kaempferol potently regresses chemoresistance in NSCLC cells (Figures 3c and g). Importantly and relevant to the clinical setting, concomitant administration of kaempferol abrogates chemoresistance to MTA in primary NSCLC cells, regardless of whether the resistance is due to an intrinsic or induced activation of the EMT pathway (Figures 4 and 5). Kaempferol-dependent abrogation of chemoresistance is likely due to the reversal of the EMT, as kaempferol treatment effectively induces a morphological switch from mesenchymal-to-epithelial phenotype and concomitantly reduces the expression of EMT-TFs and mesenchymal cell markers (Figure 3). Kaempferol belongs to the family of flavonoids that exist in many dietary plant sources and have for long time been appreciated due to multifaceted beneficial features, including cancer-preventive properties.^44^ In support of this, numerous studies have shown that kaempferol exhibits inhibitory effects on cancer cells by inducing cell cycle arrest, growth inhibition and apoptosis.^30, 31, 32^ An increasing body of evidence has also emerged that supports a crucial role for kaempferol in tumor migration and invasiveness. It has been shown that treatment of brain cancer with kaempferol (5–40 *μ*M) significantly reduces the expression of matrix metalloproteinase (MMP), a family of zinc-dependent endopeptidases that have a pivotal role in tumor metastasis.^45^ It is proposed that PKC/extracellular signal-regulated kinase (ERK)/nuclear factor (NF)-*κ*B signaling is important in regulating MMP expression and previous reports have demonstrated that treatment with 40 *μ*M kaempferol indeed inhibits the PKC/ERK/NF-*κ*B cascade.^46, 47^ Consistent with the notion that kaempferol has an inhibitory role in tumor metastasis and with our findings, a recent study shows that bronchial epithelial remodeling during airway fibrosis, in which EMT is a fundamental mechanism,^48^ can be effectively inhibited by treatment with kaempferol,^39^ and that kaempferol excels this effect by blocking TGF-*β*-dependent EMT, leading to restoration of epithelial phenotype, for example, E-cadherin expression, and suppression of mesenchymal genes such as N-cadherin.^39^

In summary, we have provided evidence that chemoresistance to MTA is driven by an activated EMT pathway and that kaempferol abrogates this chemotherapy refractory phenotype. Our findings thus provide a working model towards the development of new anticancer therapies by molecular intervention of EMT signaling and implicate the potential of kaempferol to enhance chemotherapeutic response of lung cancer.

## Materials and Methods

### Cell culture and reagents

Human NSCLC cell lines A549, H358, PC9 (adenocarcinoma) and H460 (large cell carcinoma) were obtained from ATCC (American Type Culture Collection; Manassas, VA, USA) and cultured in RPMI-1640 media (Cat. #8758; Sigma-Aldrich, St Louis, MO, USA) supplemented with 10% fetal bovine serum (FBS; Cat. #10270–106; Life Technologies, Grand Island, NY, USA) and 1% penicillin/streptomycin solution (Cat. #P0781, Sigma-Aldrich) at 37 °C in a humid atmosphere containing 5% CO_2_ and 95% air. All cell lines were DNA fingerprinted to confirm their authenticity (Microsynth, Bern, Switzerland). Pemetrexed/MTA (commercial name ‘ALIMTA' Cat. #VL7640) was purchased from Eli Lilly (Suisse) S.A. (Vernier/Geneva, Switzerland), TGF-*β* (Cat. #100-21) from Peprotech (Rocky Hill, NJ, USA), kaempferol (Cat. #420345) from Calbiochem (Darmstadt, Germany) and SB431542 (Cat. #S1067) from Selleck Chemicals (Houston, TX, USA).

The study with patient-derived primary NSCLC cells was approved by the Institutional Board of University Hospital Bern and performed according to the guidelines of the Helsinki Convention. After surgical resection at the Department of Thoracic Surgery, University Hospital Bern, the specimens were immediately transported to the Institute of Pathology, University of Bern, where a pathologist removed tumor tissue for cell culture generation. To establish primary cell culture, freshly removed NSCLC tumor tissue was washed with RPMI-1640 medium containing 1% penicillin/streptomycin, minced thoroughly into a homogeneous slurry with scalpel and subjected to collagenase digestion by resuspending in RPMI-1640 containing 4% FBS, 0.1% (g/vol) collagenase I (Cat. #LS004196; Worthington Biochemical Corporation, Lakewood, NJ, USA) and 0.25% collagenase II (Cat. #LS004176; Worthington Biochemical Corporation). The reaction was carried out for 45–60 min at 37 °C in a humidified incubator, with occasional mixing to facilitate enzymatic digestion. The digestion was stopped by adding fresh RPMI-1640 containing 10% FBS followed by filtration of the digested tissue through first 100-μm and then 40-μm strainers. Cells were collected by centrifugation at 500 × *g* for 15 min at room temperature, treated with 1 × RBC lysis buffer (eBioscience; San Diego, CA, USA) to lyse red blood cells and cultured in serum-free CnT-Prime Airway epithelial culture medium (Cat. #CnT-PR-A; CellnTec, Bern, Switzerland) supplemented with 1% penicillin/streptomycin. When reaching 80–90% confluence, the cells were passaged after detachment with TrypLE Express (Cat. #12604021, Invitrogen, Grand Island, NY, USA). Cells within the initial three passages were used for this study.

### Drug response and XTT assay

NSCLC cells were seeded in triplicate in 96-well plates, with the density of 3000 cells/well, and treated for the indicated time periods with various doses of MTA, kaemperol and vehicle (control). Cell growth and viability after treatment was assessed with XTT (3-(4,5-dimethylthiazol-2-yl)-2,5-diphenyl-tetrazolium bromide) assay (Cat. #11465015001, Roche, Germany) according to the manufacturer's instructions. The efficacy of drugs on cell growth is calculated according to NCI60 platform protocol used for human tumor cell line anticancer drug screen.^33^

### qPCR

Total RNA was isolated and purified with RNeasy Mini Kit (Cat. #74106, Qiagen, Germany). Complementary DNA (cDNA) was synthesized by the High capacity cDNA reverse transcription kit (Cat. #4368814, Applied Biosystems, Foster City, CA, USA) according to the manufacturer's instructions. qPCR analyses were performed in triplicate on a 7500 Fast Real-Time PCR System (Applied Biosystems) with commercially available TaqMan ‘Assay on Demand' primer/probes (Applied Biosystems): stem cell genes (*SOX2*, Hs 01053049_s1; *OCT4A*, Hs 03005111_g1 POU5F1; *OCT4B*, Hs 00742896_s1 POU5F1; *NANOG, Hs04260366_g1* and *ALDH1A2, Hs00180254_m1*), EMT-TFs *(SNAI1, Hs00195591_m1; SNAI2/SLUG, Hs00950344_m1; ZEB1, Hs00232783_m1; ZEB2, Hs00207691_m1),* mesenchymal markers *(CDH2/N-cadherin, Hs00983056_m1; VIM/Vimentin, Hs00185584_m1).* The gene expression level of each target gene was normalized against *GAPDH* (Hs02758991_g1) and compared among different groups by the ΔΔCT method. Baseline and threshold for Ct calculation were set automatically with the 7500 software v2.06.

### Clonogenic and sphere formation assay

Clonogenic assay was performed as described.^49^ In brief, NSCLC cells at exponential growth were treated with various reagents or FACS-sorted. Single cells were then seeded in six-well plates at a density of 100–500 cells/well. After 10–14 days depending on growth rate, the resulting colonies were stained with crystal violet (0.5% dissolved in 25% methanol) and colonies of more than 50 cells were counted.

Sphere-formation assay was performed as previously described.^43^ In brief, single-cells were cultured in sphere medium (RPMI-1640 supplemented with 20 ng/ml recombinant human epidermal growth factor (Cat. #PHG0314; Invitrogen) and basic fibroblast growth factor (Cat. #PHG0314; Invitrogen), 4 *μ*g/ml insulin (Cat. #I9278-5 ML; Sigma-Aldrich) and 1 ml B27 (Cat. #17504-044; Invitrogen)) with ultra-low attachment plates (Cat. #3471; Corning, Corning, NY, USA) for 7–10 days at 37 °C in a humid atmosphere containing 5% CO_2_ and 95% air. To optimize sphere growth, old medium was replenished by fresh medium every 3 days.

### Aldefluor assay and FACS analysis

The Aldefluor assay kit (Cat. #01700; Stem Cell Technologies, Vancouver, Canada) was used to determine the overall ALDH activity by following the manufacturer's protocol. Data acquisition was performed on LSR II flow cytometer (Becton Dickinson) based on ALDH activity, with 4-diethylaminobenzaldehyde (DEAB)-treated cells as negative control. Cells of ALDH^high^ were sorted on BD Aria using FACS Diva software (Becton Dickinson). FACS data analysis was done with Flowjo software v10 (Treestar, Oregon, USA).

### Scratch wound-healing assay

Scratch wound-healing was performed essentially as described.^50^ In brief, confluent monolayers of MTA resistant and corresponding parental cells were wounded with a plastic tip and cultured in RPMI-1640 medium without FBS or low level of FBS as indicated. Cells were observed by phase contrast microscopy at the indicated time points.

### H&E staining, Immunofluorescence and western blot analysis

Surgically removed fresh tumor tissues were formalin-fixed, paraffin-embedded, sectioned and stained with hematoxylin-eosin (H&E) using a standard protocol. For immunofluorescence, NSCLC cells grown on poly-lysine-treated coverslides were fixed with 4% paraformaldehyde for 15 min at room temperature and permeabilized with cold methanol (−20 °C) for 5 min or with 0.1% Triton X-100/PBS at room temperature for 15 min before incubated overnight at 4 °C with primary antibodies. Primary antibodies used in this study: mouse monoclonal anti-Vimentin (Cat. #ab8979; AbCam, Cambridge, UK), rabbit monoclonal anti-Vimentin (Cat. #5741; Cell Signaling Technology, Danvers, MA, USA) and mouse monoclonal anti-E-cadherin (Cat. #14472; Cell Signaling Technology). Fluorescein isothiocyanate (FITC)-conjugated anti-E-cadherin (Cat. #53-3249) and *β*-catenin (Cat. #53-2567) from eBioscience were also used. After intensive washing with 1xPBS containing 2% bovine serum albumin/BSA (Cat. #K41-001; PAA, Pasching, Austria), the cells treated with anti-Vimentin or/and anti-E-cadherin antibodies were further incubated for 1 h at room temperature with Alexa Fluor 488 goat anti-mouse IgG (Cat. #A11029), Alexa Fluor 647 goat anti-mouse IgG (Cat. #A21236) or Alexa Fluor 488 goat anti-Rabbit IgG (Cat. #A11034) from Invitrogen (Eugene, OR, USA). Nuclei were counterstained by 4′,6-diamidino-2-phenylindole. Images were acquired on a ZEISS Axioplan 2 imaging microscope (Carl Zeiss MicroImaging, Göttingen, Germany) and processed using Adobe Photoshop CS6 v.13 (Adobe Systems, San Jose, CA, USA).

For Western blot analysis, cells were lysed in RIPA buffer (Cat. #9806; Cell Signaling Technology) and equal amounts of protein lysates (15–50 *μ*g/lane) were separated by SDS-PAGE (Cat. #4561033; Bio-Rad Laboratories, Hercules, CA, USA), transferred to PVDF membranes (Cat. #170-4156; Bio-Rad), which were then blocked in blocking buffer (Cat. #927-4000; Li-COR Biosciences, Bad Homburg, Germany) for 1 h at room temperature and blotted at 4 °C overnight with primary antibodies against E-cadherin (1 : 500), Vimentin (1 : 100) and *β*-actin (1 : 20 000; Cat. #3700; Cell Signaling Technology). IRDye 680LT-conjugated goat anti-mouse IgG (Cat. #926-68020) and IRDye 800CW-conjugated goat anti-rabbit IgG (Cat. #926-32211) from Li-COR Biosciences were used in 1 : 5000 dilution. Signals of membrane-bound secondary antibodies were imaged with the Odyssey Infrared Imaging System (Li-COR Biosciences).

### Statistical analysis

All statistical analyses were performed using GraphPad Prism 6.03 (GraphPad Software, http://www.graphpad.com/welcome.htm). All data are presented as mean±S.D. from three independent experiments unless otherwise indicated. Statistical significance was determined by student's *t*-test (two tailed) and ANOVA (one-way). *P<0.05; **P<0.01 and ***P<0.001; NS, not significant.

## Abbreviations

ALDH: aldehyde dehydrogenase
CSCs: cancer stem cells
EMT: epithelial-to-mesenchymal transition
EMT-TF: epithelial-to-mesenchymal transition inducing factor
FACS: fluorescence-activated cell sorting
MPM: malignant pleural mesothelioma
MTA: multi-target folate antagonist
NSCLC: non-small cell lung cancer
qPCR: quantitative real-time PCR
SCLC: small cell lung cancer
TGF-*β*: transforming growth factor *β*
MMP: matrix metalloproteinase
ERK: extracellular signal-regulated kinase
NF: nuclear factor
FBS: fetal bovine serum
