# Supplementary material for: Blocking the epithelial-to-mesenchymal transition pathway abrogates resistance to anti-folate chemotherapy in lung cancer
Source: Cell Death Dis. 2015 Jul 16;6(7):e1824–. doi: 10.1038/cddis.2015.195 (PMC4650740; doi:10.1038/cddis.2015.195)
Supplement: Supplementary Information [file cddis2015195x1.pdf]

SUPPLEMENTARY INFORMATION

Supplementary Figure S1 Chemoresistance to MTA in NSCLC cells. **(A-B)** A549, H358 and H460 cells treated with the indicated doses of MTA for different time periods were analyzed by XTT assay. MTA effect on cell growth was calculated according to the NCI60 platform protocol. Results are presented as mean ± SD of three independent experiments (n=3). Note that the proliferative potential of a subset of tumor cells in all three NSCLC cell lines persisted with high MTA doses and prolonged treatment. GI50, 50% growth inhibition; TGI, total growth inhibition; LC50, 50% lethal concentration.

Supplementary Figure S2 Acute MTA treatment induces clonogenic potential. (**A-C**) A549, H460 and H358 cells treated with the indicated MTA doses (5.0 and 1.0 µM) and time periods (0, 6 and 12 hours) were seeded in 6-well plates (250 cells/well) and cultured for 10 days (A549, H460) or 14 days (H358) for colony formation. Data are shown as mean ± SD of three independent experiments (n=3).

Supplementary Figure S3 Acute MTA treatment induces ALDH expression and augmented ALDH activity features a stem cell-like phenotype. (**A**) A549, H358 and H460 cells treated with MTA (10µM) or vehicle for 24h were subjected to Aldefluor assay and analyzed by flow cytometry for ALDH expression. Aldefluor, substrate of ALDH; DEAB, inhibitor of ALDH. (**B**) The gating strategy used for FACS-based sorting of the $ALDH^{high}$ subset from bulk tumor cells. (**C**) The mRNA level of stem cell factors (Sox2, Oct4, Nanog)

in ALDH^{high} (H358 ALDH^{high}) and unsorted H358 cells was analyzed by qPCR. Data are shown in mean ± SD of three independent experiments (n=3). (**D**) Comparison of clonogenic potential between H358 ALDH^{high} and unsorted H358 cells. Cells were seeded in 6-well plates (250 cells/well) and cultured for 14 days for colony formation. Data are shown in mean ± SD of three independent experiments (n=3). (**E**) The mRNA level of stem cell factors in ALDH^{high} (H460 ALDH^{high}) and unsorted H460 cells were analyzed by qPCR. Data are shown in mean ± SD of three independent experiments (n=3). (**F**) Comparison of clonogenic potential between H460 ALDH^{high} and H460 cells. The cells were seeded in 6-well plates (250 cells/well) and cultured for another 10 days for colony formation. Data are shown in mean + SD of three independent experiments (n=3). (**G**) *ALDH1A2* is a prognostic marker in NSCLC. Meta-analysis of *ALDH1* isoforms (A1, A2 and A3) in a cohort of 1570 samples showed that high levels of *ALDH1A2* correlated with poorer prognosis in NSCLC.

Supplementary Figure S4 MTA resistant NSCLC cells exhibit an activated EMT phenotype. (**A, B**) Phase contrast images of MTA resistant H358_R, H460_R and their parental H358 and H460 cells. Scale bar, 50 μm. (**C**) Western blot analyses for E-cadherin and Vimentin in MTA resistant (A549_R, H460_R and H358_R) and the corresponding parental cells. β-actin was used as loading control and the positions of molecular mass markers were indicated to the left. (**D, E**) MTA resistant cells show higher mobility than their parental cells. Scratch wound-healing assay showing that monolayers of A549, A549_R (D), H358 and H358_R (E) were wounded with plastic tips, cultured in RPMI-1640 medium supplemented with 1% FBS (A549 and A549_R) or in serum-free medium (H358 and H358_R) and monitored at the indicated time points. Scale bar, 100 μm

Supplementary Figure S5 TGF-β induces EMT and kaempferol reverses the phenotype. Cells of H358, H358 with an activated EMT after TGF-β treatment (H358_EMT) and the H358_EMT treated with kaempferol (20µM) for 5 days (H358_EMT+Kae) were analyzed by immunofluorescence for E-cadherin (red) and Vimentin (green). Nuclei were counterstained with DAPI (blue). Scale bar, 25 µm.

Supplementary Figure S6 TGF-β induces clonogenic and chemoresistant potential in NSCLC cells. (**A**) A549 cells treated with 5ng/ml TGF-β (A549+TGF-β) or vehicle (A549) for 14 days were analyzed by qPCR. (**B, C**) H358 cells treated with 5ng/ml TGF-β (H358+TGF-β) or vehicle (H358) for 14 days were seeded in 6-well plates (250 cells/well), cultured for 14 days and analyzed by clonogenic (B) and sphere formation (C) assay. (**D**) TGF-β- and vehicle-treated A549 cells (as above) were seeded in 6-well plate (250 cells/well), cultured for 10 days and analyzed by clonogenic assay. (**E**) TGF-β- and vehicle-treated A549 cells (as above) were exposed to various MTA doses for 5 days. Drug response to MTA was determined by XTT assay. All the results are shown in mean + SD of three independent experiments (n=3).

Supplementary Figure S7 Kaempferol and SB431542 reverse EMT phenotype and regress resistance to MTA. (**A**) MTA resistant H358_R cells treated with vehicle (upper panel) or kaempferol (20µM) for 3 days (lower panel) were immunostained with antibodies against E-cadherin (red) and Vimentin (green). Nuclei were counterstained with DAPI (blue). Scale bar, 25 µm. (**B**) Kaempferol and SB431542 abrogate chemoresistance to MTA. A549_R and H358_R cells treated with vehicle, kaempferol (20µM) or SB431542

(40µM) for 3 days were exposed for an additional 5 days to MTA. Drug response was determined by XTT assay and data are shown as mean ± SD of three independent experiments (n=3).

Supplementary Figure S8 Micrographic images of tumor specimens that were used for the establishment of primary NSCLC cells. Surgically resectioned fresh tumor samples were fixed in formalin and embedded in paraffin using a standard protocol. Slides were stained with hematoxylin and eosin. Images were acquired on a ZEISS Axioplan 2 imaging microscope at an objective magnification of x10.

Supplementary Figure S9 TGF-β and MTA induces an epithelial-mesenchymal phenotype in primary NSCLC cells. Primary BE088T cells (BE088T) and BE088T cells treated for 14 days with 5ng/ml TGF-β (BE088T+TGF-β) or with 5µM MTA (BE088T_R) were immunostained with antibodies against E-cadherin (red) and Vimentin (green). Nuclei were counterstained with DAPI (blue). Scale bar, 25 µm.

Supplementary Table S1 Relative EMT gene expression in NSCLC cell lines

**NSCLC cell line /Gene**	***SNAI1***	***SNAI2***	***ZEB1***	***ZEB2***	***Vimentin***
PC9	1	1	1	1	1
H358	5.5	1.7	7.2	0.6	50.3
A549	5.3	0.03	88.6	8.3	211.5
H460	4.3	0.44	62.1	121.6	210.9

Supplementary Table S2 Clinicopathological characteristics of NSCLC patients analyzed in this study

Patient ID	Age/sex	Histology type	Neoadjuvant treatment	Stage
**BE060**	57/f	NSCLC/ squamous cell carcinoma, G3	None	IIB (pT2bN1M0)
**BE069**	65/m	NSCLC/ adenocarcinoma, G3	None	IIA (pT1bN1M0)
**BE079**	73/f	NSCLC/ squamous cell carcinoma, G3	None	IIIA (pT3N1M0)
**BE084**	73/f	NSCLC/ squamous cell carcinoma	4 cycles Cisplatin and Navelbine (08-11/2013)	IIIA (ypT4N1M0)
**BE088**	49/m	NSCLC/ squamous cell carcinoma, G2	None	IB (pT2aN0M0)
**BE090**	73/m	NSCLC/ squamous cell carcinoma, G3	None	IIA (pT2bN0)

Supplementary Table S3 Relative EMT gene expression in human primary NSCLC cells

Primary culture / Gene	*SNAI1*	*SNAI2*	*ZEB1*	*ZEB2*	*Vimentin*
**BE088T**	1	1	1	1	1
**BE079T**	40.3	0.57	251.4	1482.2	12.5
**BE069T**	297.4	0.45	1094.6	3338.1	7.3
**BE084T**	101.0	0.5	506.7	1425.3	6.2
**BE090T**	54.3	0.87	431.0	2559.2	7.8
**BE060T**	76.2	0.60	407.3	4414.5	10.0

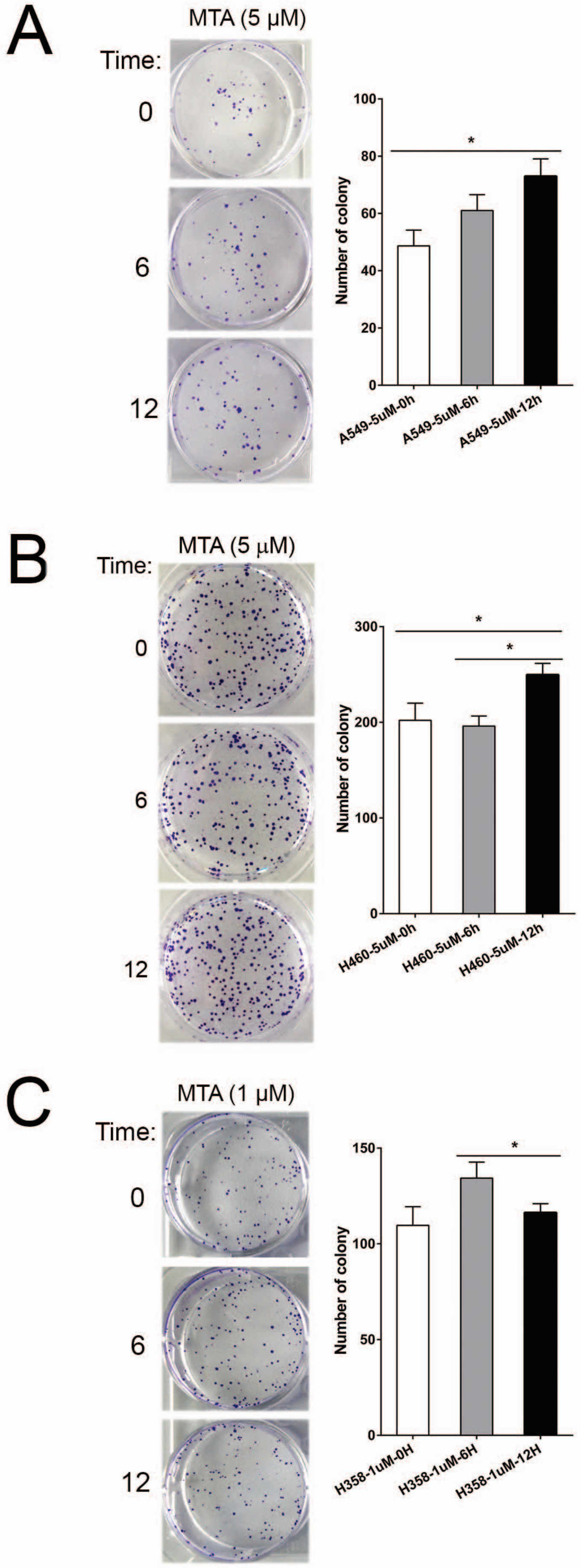

Liang *et al.*, Supplementary Figure S2

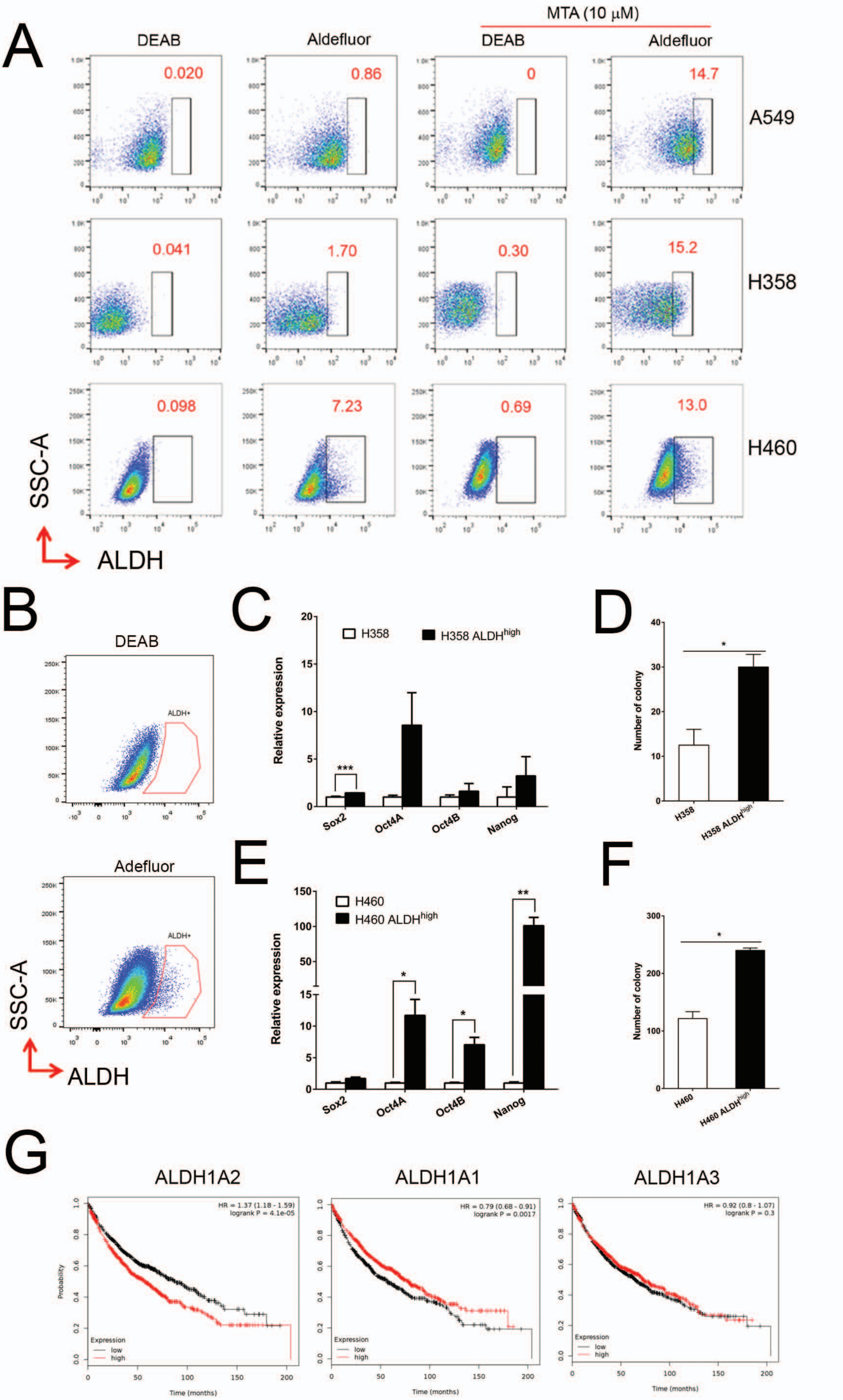

Liang *et al.*, Supplementary Figure S3

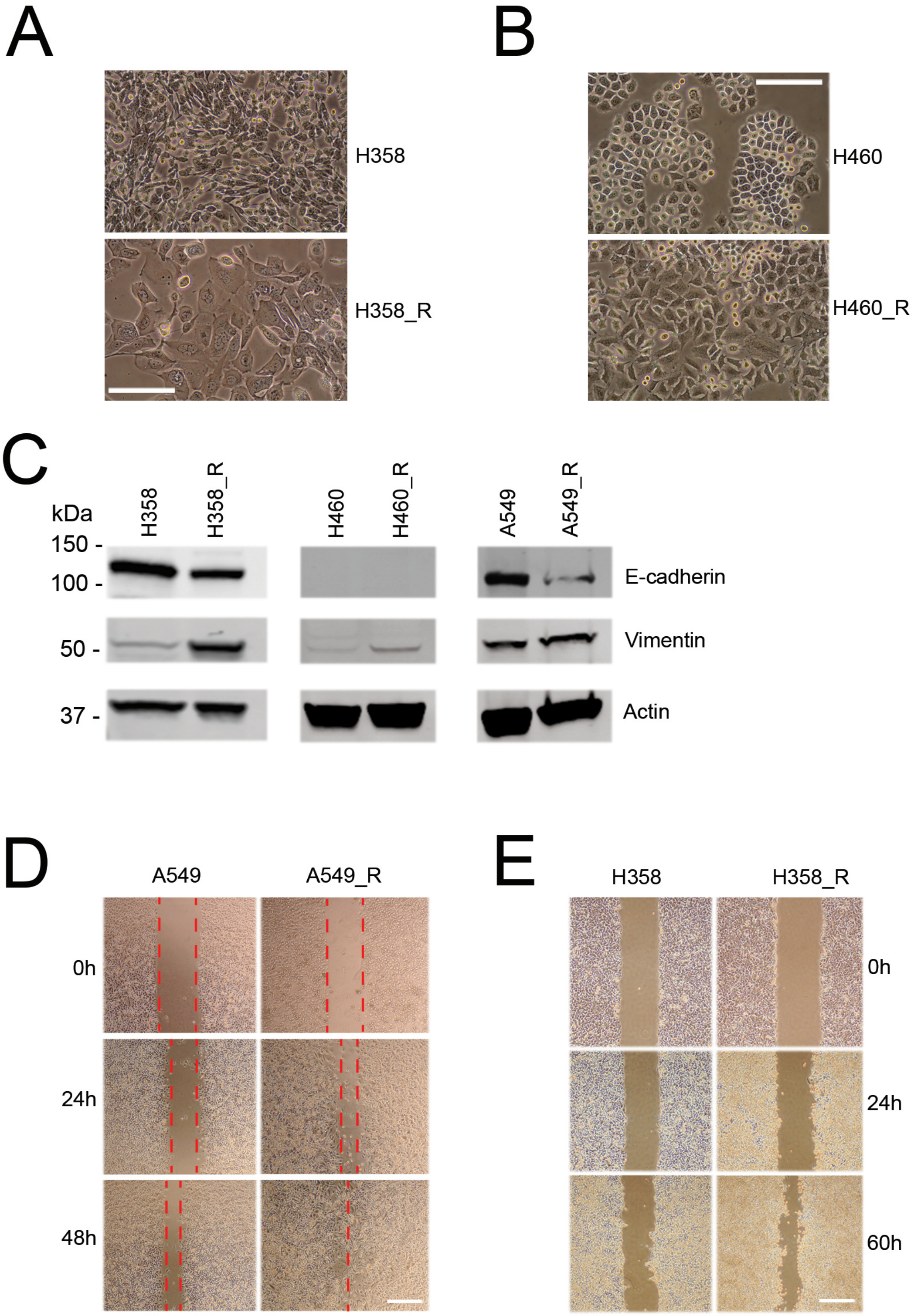

Liang *etal.*, Supplementary Figure S4

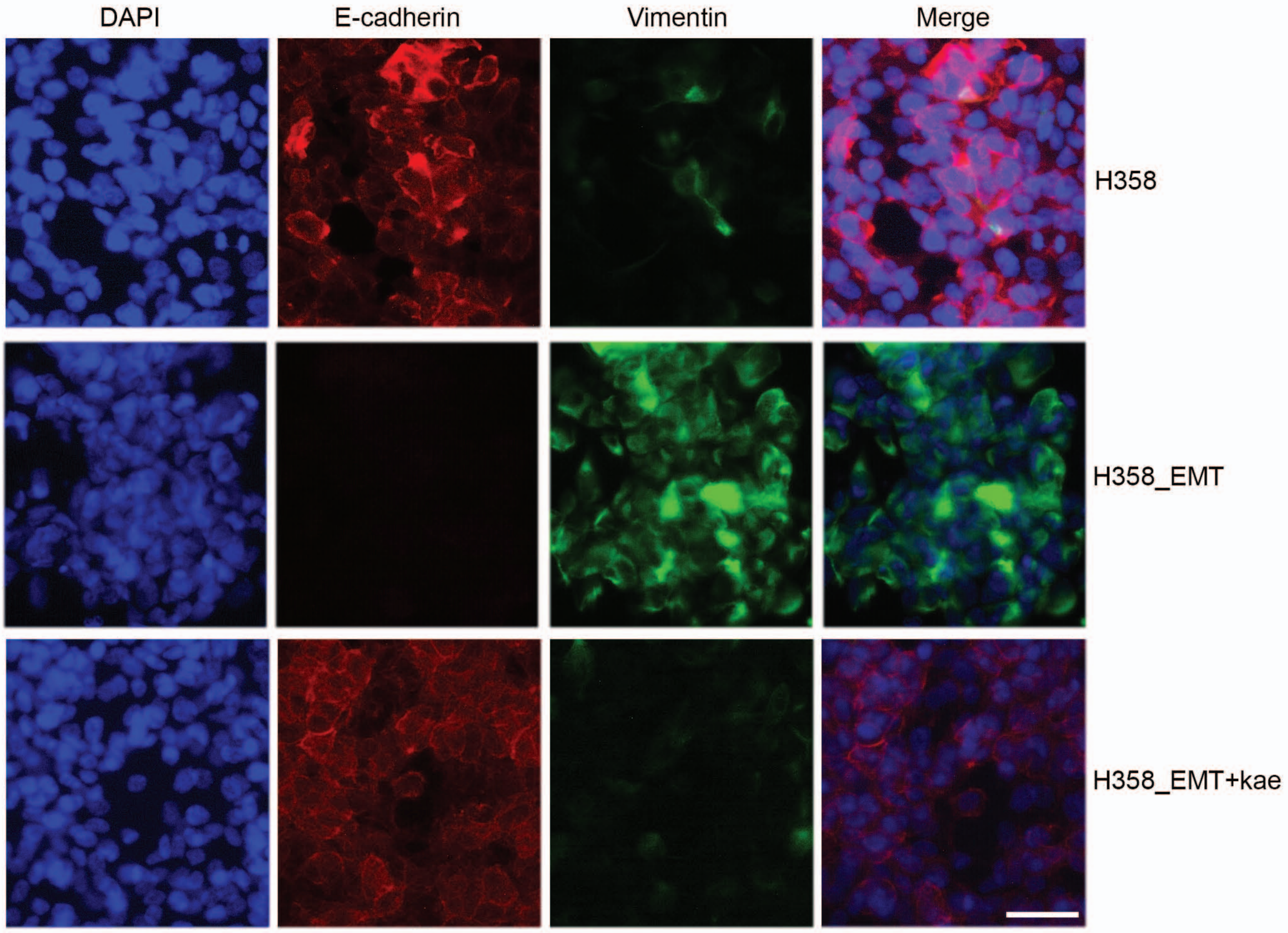

Liang *et al*., Supplementary Figure S5

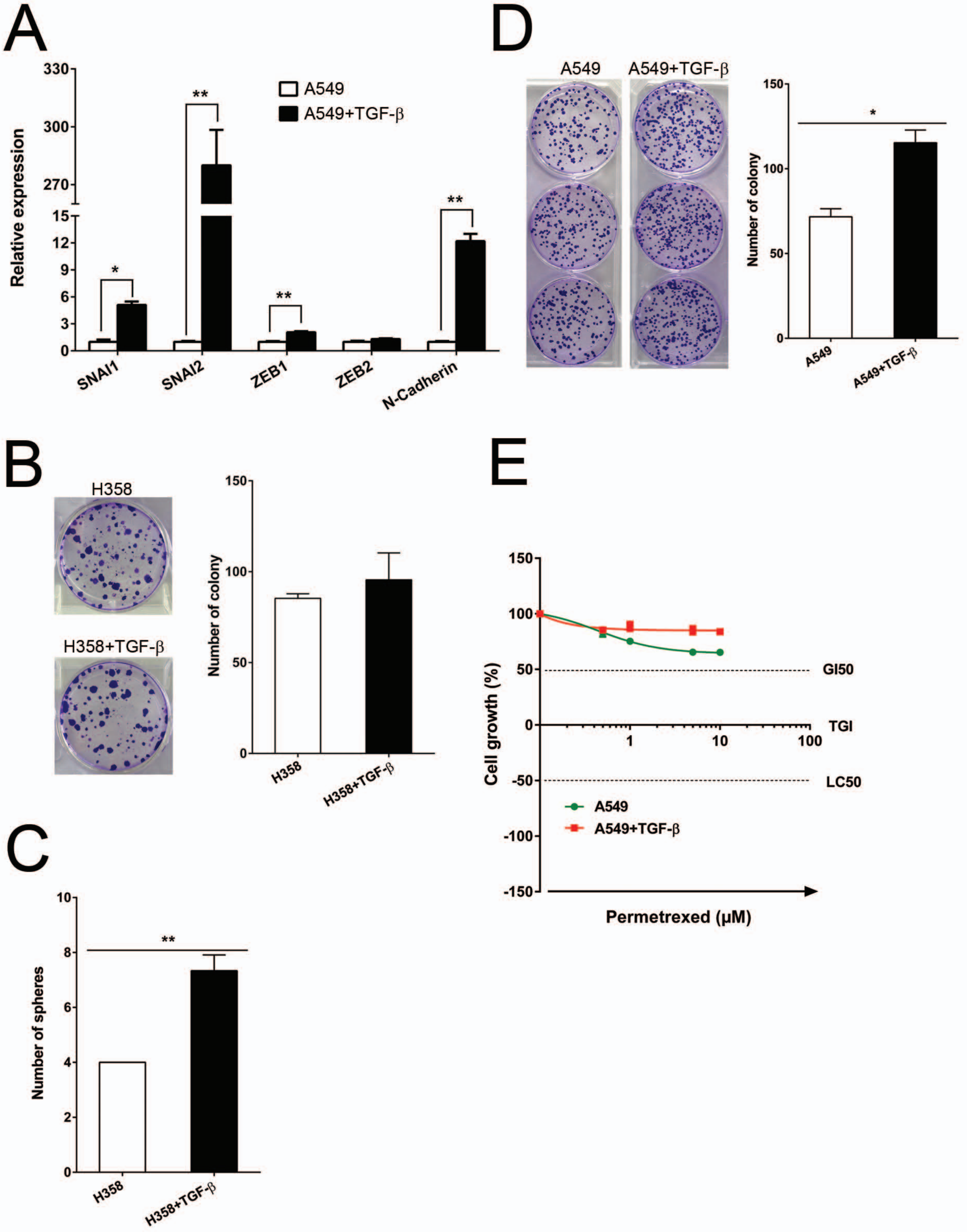

Liang *et al*., Supplementary Figure S6

A

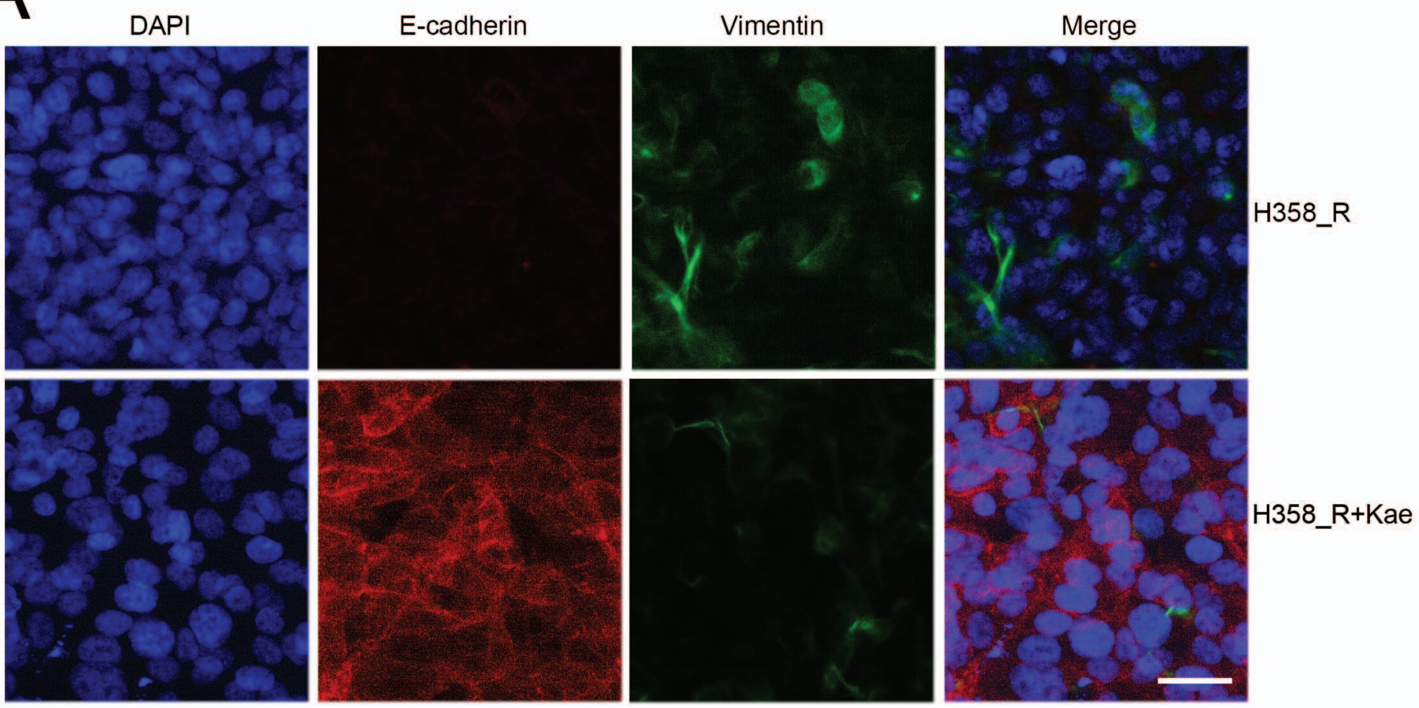

B

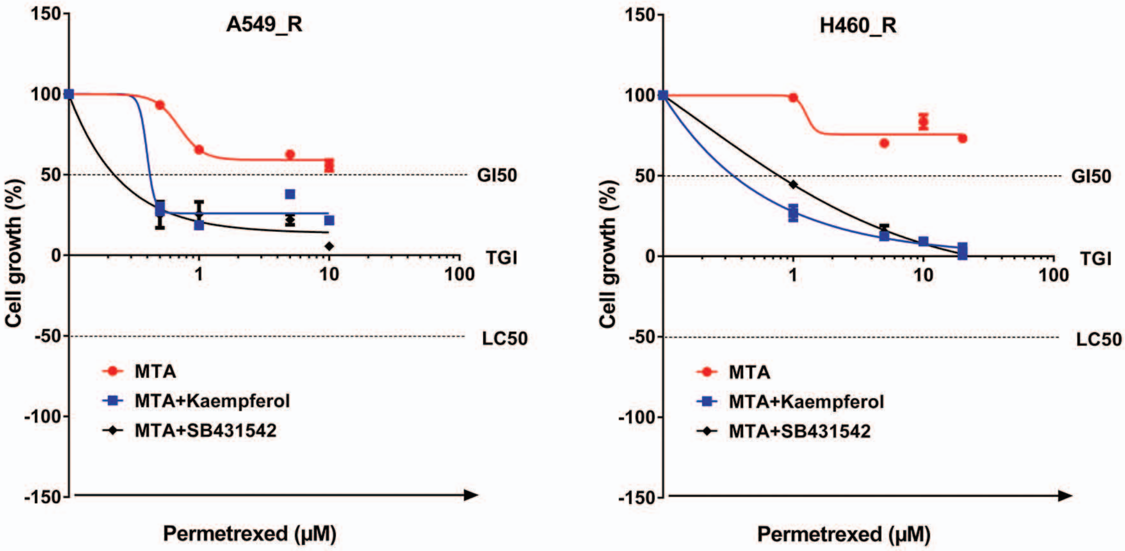

Liang *et al.*, Supplementary Figure S7

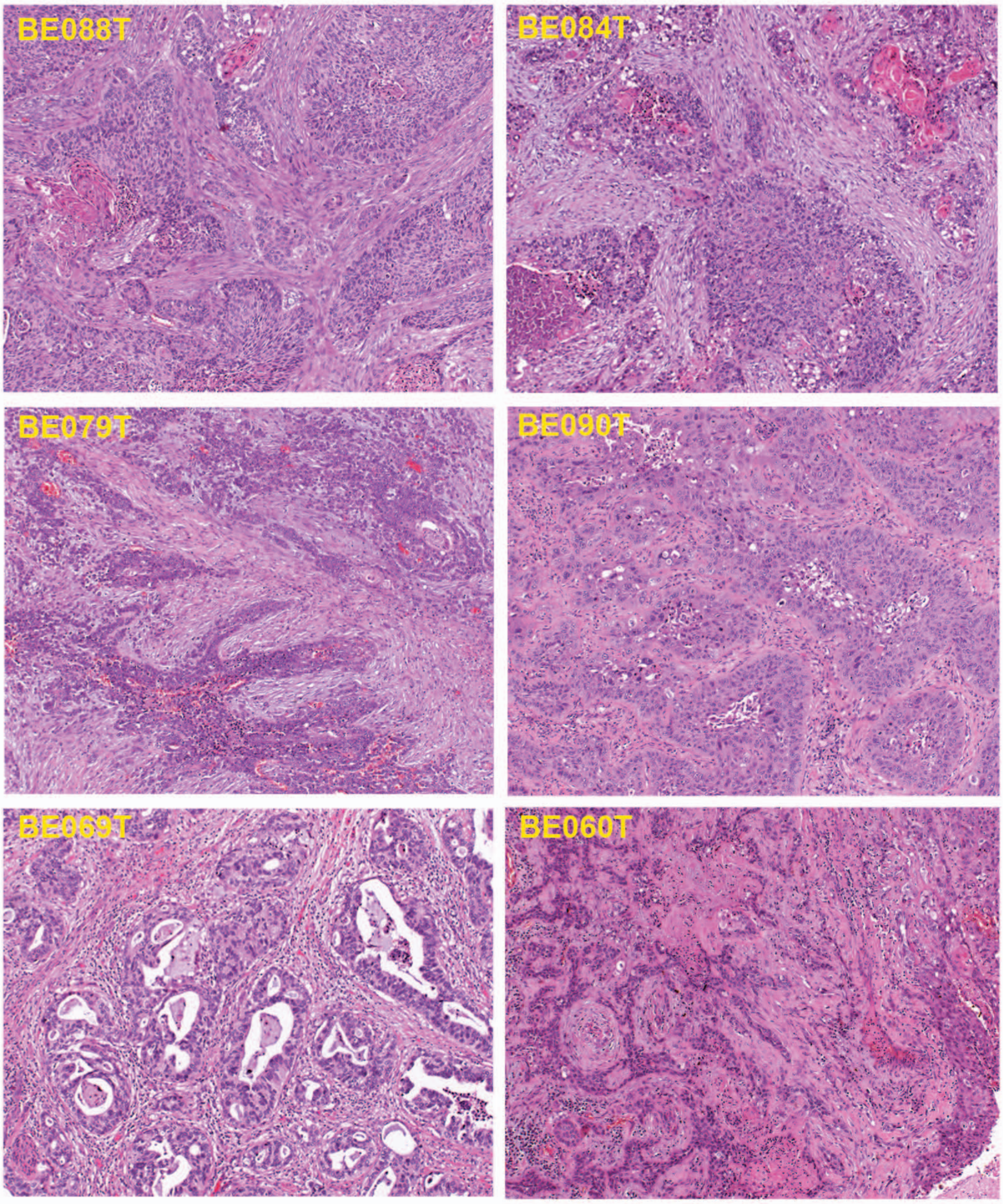

Liang *et al.*, Supplementary Figure S8

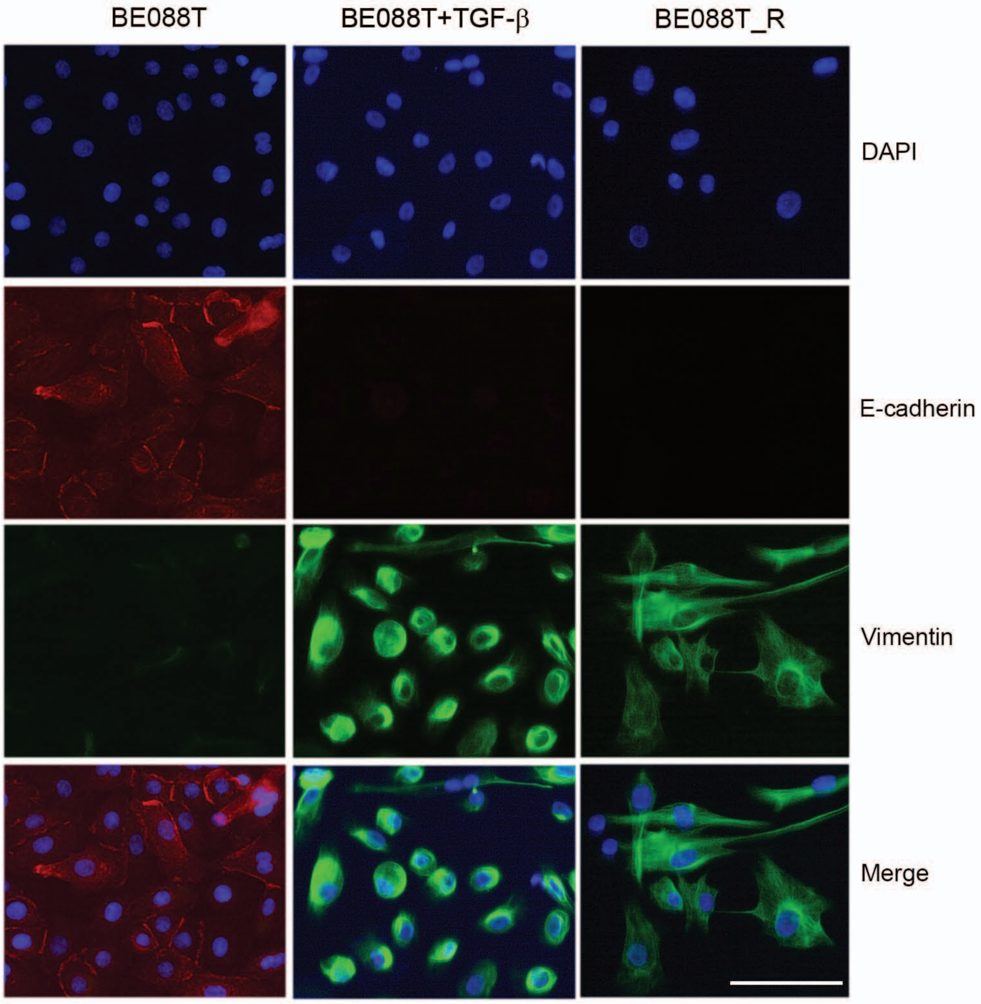

Liang et al., Supplementary Figure S9
